# Blood lead concentration and its associated factors in preschool children in eastern Iran: a cross-sectional study

**DOI:** 10.1186/s12887-020-02302-7

**Published:** 2020-09-16

**Authors:** Mahmoud Zardast, Seyedeh Samira Khorashadi-Zadeh, Samaneh Nakhaee, Alireza Amirabadizadeh, Omid Mehrpour

**Affiliations:** 1 Medical Toxicology and Drug Abuse Research Center (MTDRC), Birjand University of Medical Sciences, Birjand, Iran; 2 Student Research Committee, Birjand University of Medical Sciences, Birjand, Iran; 3 Cardiovascular Diseases Research Center, Birjand University of Medical Sciences, Birjand, Iran; 4 Arizona Poison & Drug Information Center, the University of Arizona, college of pharmacy, Tucson, AZ USA

**Keywords:** Blood lead concentration, Children, Iran, Lead toxicity

## Abstract

**Background:**

Lead is a toxic metal that affects almost every organ in the body. Children are more susceptible to lead toxicity because they ingest non-food items (pica), have oral exploratory habits, absorb more substantial amounts of ingested lead compared to adults, and have a developing central nervous system. This study describes venous blood lead concentrations (BLC) in young children living in Birjand, Iran.

**Methods:**

A cross-sectional study was performed in 2016 on children 1–7 years of age who were referred to healthcare centers in Birjand City. Demographic information was obtained, and their BLC was tested using atomic absorption spectrometry (AAS).

**Results:**

Four hundred children were tested. Their mean age was 52.37 ± 23.77 months; their mean BLC was 2.49 ± 2.64 μg/dL (median 1.85 μg/dL). Thirty-two (8%) children had a BLC > 5 μg/dL. A logistic regression model revealed that per one unit of increase in age, the chance of an elevated BLC decreased by 3% (OR (95%CI): 0.97 (0.96–0.99), *p* < 0.01). The risks of an elevated BLC was 61% lower in girls compared to boys (OR (95%CI): 0.39 (0.17–0.92), *p* = 0.03). Further, per one rate of increase in the BMI, the chance of an elevated BLC was higher (OR (95%CI): 1.13 (1.02–1.24), *p* = 0.01). Children whose fathers were laborers had higher BLC than those with employee fathers (*p* = 0.01).

**Conclusion:**

Of 400 children aged 1–7 years old living in Birjand, Iran, 8% had elevated BLC. BLC correlated with the child ‘s age, gender, body mass index, and father’s occupation.

## Background

Lead is a toxic environmental metal that influences almost every organ in the body [1]. Lead toxicity may happen in children because they ingest non-food items (pica), have oral exploratory habits, absorb more substantial amounts of ingested lead compared to adults, and they are more vulnerable to lead toxicity because they have a developing central nervous system [2, 3]. Lead is associated with numerous complications in children, such as neurological and neurobehavioral defects, lowered intelligence quotient (IQ), and developmental disorders [4]. Even low blood lead concentrations (BLC) can have detrimental effects on the ability of children in learning, attention, and productivity at school [5]. Blood lead concentrations have a short biological half-life that indicates the dynamic interaction among absorption, excretion, and transfer to and reabsorb from other body compartments. Thus, lead effects may not occur at well-defined blood lead concentrations [6], and there is no safety threshold for BLCs [7]. Further, the blood lead concentration may not precisely reflect the accumulation of tissue lead, and toxicity may happen at a low blood lead concentration. Thus, the effects of lead toxicity may have been found at blood lead concentrations below the recognized “safe” limits [6]. However, the Centers for Disease Control and Prevention (CDC) established the reference BLC as 5 μg/dL or less for children [8].

In 1991, CDC highly recommended screening by a blood lead test at least once for all children younger than 2 years of age [9]. Children may be exposed to lead in air, food, water, dust and debris, gasoline, dyes, and other products such as some types of traditional medicine [7]. Prior studies have shown contamination of soil, water, and some food of our region (Birjand, Iran) with different metals, including lead [10–12]. Birjand is the capital of South province in the east of Iran. It is one of the economically deprived areas of the country characterized by low income, inappropriate residential houses, lack access to clean water in some areas. Agricultural section and animal husbandry play an essential role in the economy of the province and particularly in rural regions of the province, and the life of people is dependent on it. While screening the BLC of children is practiced in other countries, no study has investigated the BLC in young children exposed to lead in the southern Khorasan province of Iran. In this study, we investigated the BLCs and its associated factors (demographic factors such as age, sex, etc.; social parameters such as occupation and education levels of parents, addiction and cigarette smoking in parents and etc.; essential trace elements such as calcium, magnesium, iron, and zinc concentrations; and hematological indices) in young children (1–7 years) living in Birjand, Iran. Also, this cross- sectional study tested the hypothesis, which children with low social status / with parental exposure more likely to have elevated lead concentrations.

## Methods

In this descriptive and analytical study, 400 children with 1–7 years of age in Birjand city were studied during September to December 2016. The primary outcome was elevated blood lead concentrations (BLC ≥ 5 μd/dL) and some potential associated factors of BLCs in children. Birjand city was divided into four parts based on the four major healthcare centers in this city (the capital of southern Khorasan province in eastern Iran). Primary Health Care Centers is one of the health care networks in Iran run by the Ministry of Health and Medical Education (MOHME). These centers are a far-reaching network of public clinics that provide primary and preventive health care such as nutrition, family planning, hypertension assessment, prenatal care, immunization, and environmental monitoring. These centers are the first level of communication between families and the health system. Children in this study typically go to health centers and use health centers in the province.

A total sample size of 400 children was determined based on the study of Wang et al., With a first error level of 5%, a standard deviation of 0.15 and accuracy of 10% of standard deviation error [13].

**Fig. 1:**
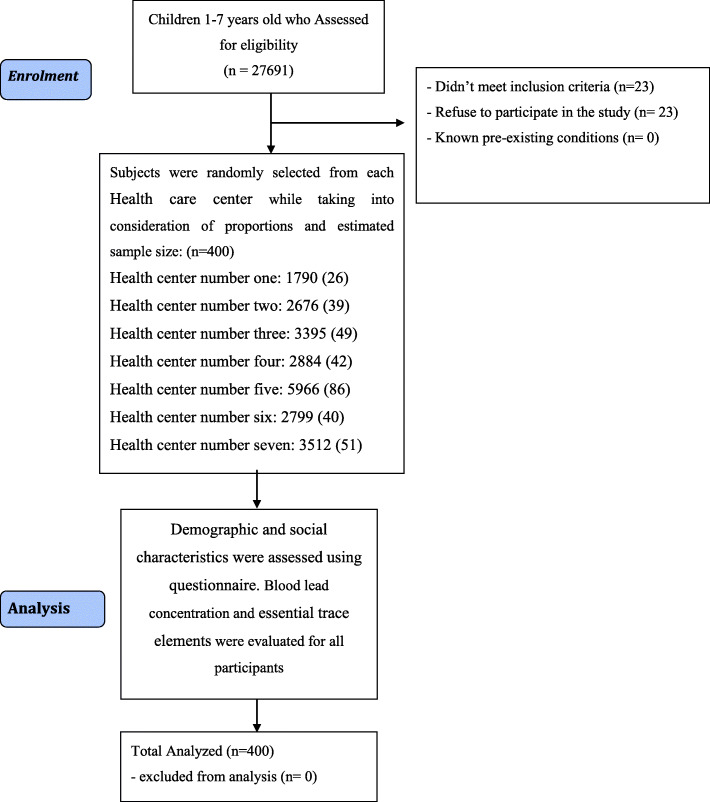
Flow diagram of children’ enrolment throughout the study

From each healthcare center, a health base was chosen through simple random sampling from the list of the healthcare bases covered by each center. Then, 1-to-7-year-old children were ascertained through simple random sampling in line with the sample size from each base. The total number of children aged 1–7 years, covered by Birjand Health Centers, is 27,691 people. The selected number of children and the total number of children covered by each Health center of 1–8 in Birjand city were 1790(26), 2676(39), 3395 (49), 2884 (42), 5966 (86), 2799 (40), 3512 (51), 4669 (67), respectively (Fig. 1). Inclusion criteria were age 1–7 years old, consent of parents for participation in the study, subjects without identified pre-existing illness. Twenty-three parents refused to participate in the study.

Before a child was recruited into the study, informed consent was obtained from the parents. A questionnaire consisting of 22 items about demographics, as well as the risk factors of lead exposure, was then administered to parents. The obtained information was demographic factors such as age, sex, etc.; social parameters such as occupation and education levels of parents, addiction and cigarette smoking in parents, exposure to soil, and paint and etc.; essential trace elements such as calcium, magnesium, iron, and zinc concentrations; and hematological indices.

In this study, different occupations were defined as bellow:

Employee: A person who works full-time or part-time under a contract of employment, working for an organization, company, or community and has recognized rights and duties.

Laborer: a person is doing unskilled manual work for wages.

Self-employment: the state of working for oneself rather than an employer. Self-employed persons, or independent contractors, earn income by contracting with a trade or business directly. The self-employed do not receive benefits such as health insurance. Their salaries are not constant, and they have variable working hours.

### Laboratory measures

5–10 mLs venous blood was taken from each child by forearm venipuncture using needles and vacuum tubes by a trained nurse; part of it was mixed with EDTA, and the other part was blended with heparin (20 units for each ml of blood) and sent to Imam Reza Hospital laboratory. The blood containing EDTA was used for performing a complete blood count (CBC) using the Cell Dyn Analyzer. Blood calcium, magnesium, iron, and zinc concentrations were measured using a Selectra automatic device. In order to collect the samples for lead concentrations, the free sodium heparin pink-cap 6-mL Vacuette tubes were applied. Then the samples in ice containing Styrofoam boxes were transported to the laboratory. The lead concentration in blood was measured using an atomic absorption spectrometer (Varian Co. AA500AFG, Leicester City, UK), using the standards recommended by the National Institute of Occupational Safety and Health (NIOSH) [14].

### Data analysis

Data analysis was conducted through SPSS 19. Descriptive indices, including frequency, percentage, mean, and standard deviation, were calculated. Using the Kolmogorov Smirnov test, the assumption of normality of quantitative variables was tested. Mann-Whitney and Kruskal Wallis tests were used to compare BLC with demographic variables. With a logistic regression model, the factors possibly associated with BLC were inspected. Pearson correlation coefficient tests were employed to investigate the relationship between variables. An alpha level of significance was set at 0.05.

### Investigational review board approval

The research proposal was approved by the research deputy of Birjand University of Medical Sciences and by the ethics committee (ir.bums. REC.1395.131). Children with elevated lead concentrations who need treatment (*n* = 1) were followed and received free medical care.

## Results

Data on 400 children aged 1–7 years in Birjand City were collected in 2016. A total of 400 subjects were enrolled that were included among the total children (*n* = 27,691) that routinely refer to Health Care centers. Their mean age was 52.37 ± 23.78 months (range: 12 to 116 months); 200 (50%) of the children were boys. The mean BLC was 2.49 ± 2.64 μg/dL (range: 0.5–29.40 μg/dL) with a median of 1.85 [IQR: 1.10–2.87] μg/dL (Table 1). The mean BLC in males was 2.40 ± 2.85 μg/dL (median 1.90 [IQR:1.10–3.20] ug/dL) and in females, it was 2.58 ± 2.40 μg/dL (median 1.70 [IQR:1.10–2.80] μg/dL) (Table 2). The results of the Mann-Whitney test showed that the lead concentration was not significantly different between boys and girls (z = 0.86, *p* = 0.39). The mean lead concentration in those whose father was laborer was 3.20 ± 3.96 with a median of 1.80 [IQR:1.20–3.30] μg/dL, and in drivers, it was 1.79 ± 0.87 with a median of 1.70 [IQR: 1.00–2.30] μd/dL. The results of the Kruskal Wallis test indicated that the lead concentration was not significantly different between fathers with different occupations (X2 = 3.25, *p* = 0.66). Twelve (3%) family members had a history of drug abuse. The mean BLC in subjects whose family members had a history of drug use was 2.43 ± 1.53 μg/dL (median 2.1 [IQR: 1.17–3.12] μg/dL). The mean BLC in the parents using opium was 2.95 ± 2.10 μg/dL with a median of 2.45 [IQR: 1.25–3.18] ug/dL, in industrial drugs of abuse, it was 1.67 ± 0.61 with the median of 1.65 [IQR: 1.02–2.86] μd/dL. The mean BLCs in children exposed to paint was 2.92 ± 3.32 μg/dL (median 2.10 [IQR: 1.20–3.30] μg/dL) (Table 2).

**Table 1:** Frequency distribution of demographic information of children under study

Variable	Mean ± SD/frequency (percentage)
Age (month)	52.37 ± 23.78
Body mass index (kg/m^2^)	15.52 ± 4.13
Gender	
Boy	200 (50%)
Girl	200 (50%)
BLC	2.49 ± 2.64 (range: 0.5–29.40 μg/dL)
< 2.5 μg/dL	273 (68.3%)
2.5–5 μg/dL	95 (23.7%)
5–7.5 μg/dL	17 (4.2%)
7.5–10 μg/dL	6 (1.5%)
> 10 μg/dL	9 (2.3%)

**Table 2:** Relationship between different variables and blood lead concentrations of study subjects

Variable	Mean ± SD	Median [IQR]	Test result
Gender			
Boy	2.40 ± 2.85	1.90 [1.10–3.20]	z = 0.86 *p* = 0.39
Girl	2.58 ± 2.40	1.70 [1.10–2.80]	
Age			
≥ 72 month	3.03 ± 2.28	2.40 [1.10–4.60]	χ^2^ = 5.58 *p* = 0.13
12–24 month	2.86 ± 4.15	1.85 [1.10–2.60]	
24–48 month	2.08 ± 1.34	1.70 [1.10–2.50]	
48–72 month	2.28 ± 2.19	1.70 [1.10–2.80]	
Exposure to paint			
Yes	2.92 ± 3.32	2.10 [1.20–3.30]	z = 1.39 *p* = 0.16
No	2.39 ± 2.46	1.80 [1.10–2.75]	
Exposure to soil			
Yes	2.49 ± 1.61	2.20 [1.10–3.25]	z = 1.02 *p* = 0.31
No	2.48 ± 2.75	1.80 [1.10–2.70]	
Occupation of father			
Employee	2.18 ± 1.33	1.90 [1.10–2.80]	χ^2^ = 3.25 *p* = 0.66
Martial	2.82 ± 2.10	2.10 [1.10–4.15]	
Laborer	3.20 ± 3.96	1.80 [1.20–3.30]	
Repairman	2.30 ± 1.20	2.25 [1.35–2.97]	
Driver	1.79 ± 0.87	1.70 [1.00–2.30]	
Self-employment	2.42 ± 2.85	1.10 [1.70–1.70]	
Education levels of parents			
Primary school	3.04 ± 3.89	1.60 [1.10–3.20]	χ^2^ = 0.87 *p* = 0.83
Middle School	2.46 ± 1.64	2.05 [1.12–3.30]	
Diploma	2.51 ± 2.96	1.80 [1.10–2.75]	
Bachelor	2.16 ± 1.43	1.80 [1.10–2.80]	
Occupation of mother			
Housewife	2.45 ± 2.64	1.80 [1.10–2.80]	χ^2^ = 1.79 *p* = 0.41
Employment	2.47 ± 1.61	1.90 [1.12–3.42]	
Self-employment	3.29 ± 4.26	2.05 [1.40–3.32]	
Opium use in parents			
Yes	2.43 ± 1.53	2.10 [1.17–3.12]	z = 0.64 *p* = 0.52
No	2.48 ± 2.66	1.80 [1.10–2.80]	
Cigarette smoking			
Yes	2.49 ± 2.70	1.90 [1.20–3.17]	z = 0.58 *p* = 0.56
No	2.32 ± 1.41	1.80 [1.10–2.80]	

In children with BLC above 5 μg/dL, 15 (46.8%) had anorexia, 3 (9.4%) had persisted vomiting, 2 (6.3%) had constipation and abdominal pain, and 8 (25%) were asymptomatic. The results of the Chi-Square test showed that there was no significant relationship between BLC and clinical symptoms (*p* = 0.12) (Table 3).

**Table 3:** The association of elevated blood lead concentrations with clinical symptoms in study subjects

Variable	Total	BLC ≥ 5	BLC < 5	Test result
Anorexia	100 (25.0%)	15 (46.8%)	85 (23.1%)	*P* = 0.07
Weight Loss	25 (6.3%)	1 (3.1%)	24 (6.5%)	*P* = 0.10
Paleness	11 (2.7%)	1 (3.1%)	10 (2.7%)	*P* = 0.08
Constipation	14 (3.6%)	2 (6.3%)	12 (3.2%)	*P* = 0.19
Abdominal pain	17 (4.2%)	2 (6.3%)	15 (4.2%)	*P* = 0.06
Vomiting	23 (5.7%)	3 (9.4%)	20 (5.4%)	*P* = 0.25
Ataxia	0 (0%)	0 (0%)	0 (0%)	*P* = 0.99
Seizure	0 (0%)	0 (0%)	0 (0%)	*P* = 0.93
Speech defect	0 (0%)	0 (0%)	0 (0%)	*P* = 0.99
Memory defect	0 (0%)	0 (0%)	0 (0%)	*P* = 0.99
Asymptomatic	210 (52.5%)	8 (25.0%)	202 (54.9%)	*P* = 0.06

**Fig. 2:**
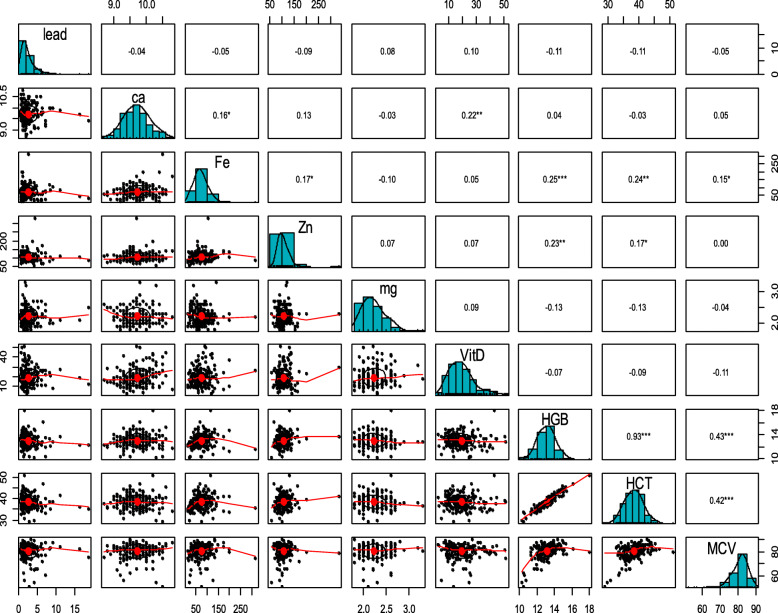
Investigating the correlation between the BLCs and calcium, iron, zinc, magnesium, hemoglobin, hematocrit, MCV, and vitamin D in boys. BLC (μg/dL); Ca (mg/dl); Fe (mg/dl); Zn (mg/dl); Mg (mg/dl); Vit D (ng/ml); HGB (g/dl); HCT (%); MCV (FL). *: correlation is significant at the 0.05 level. **: correlation is significant at the 0.01 level. ***: correlation is significant at the 0.001 level

**Fig. 3:**
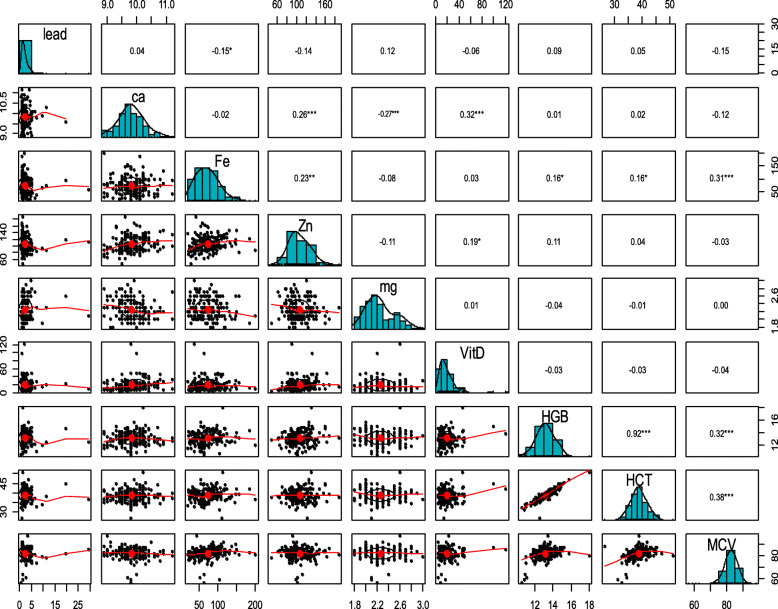
Investigating the correlation between the BLC and calcium, iron, zinc, magnesium, hemoglobin, hematocrit, MCV, and vitamin D in girls. BLC (μg/dL); Ca (mg/dl); Fe (mg/dl); Zn (mg/dl); Mg (mg/dl); Vit D (ng/ml); HGB (g/dl); HCT (%); MCV (FL). *: correlation is significant at the 0.05 level. **: correlation is significant at the 0.01 level. ***: correlation is significant at the 0.001 level

The results of Spearman correlation coefficient tests showed no significant relationship among boys between the BLC and iron, calcium, zinc, magnesium, vitamin D, hemoglobin, hematocrit, and MCV (Fig. 2). However, in girls, there was an inverse and significant relationship between lead and iron concentrations (*r* = − 0.15, *p* = 0.04) (Fig. 3).

The regression model indicated that the variables of age, gender, and BMI significantly increase the probability of having BLC > 5 μg/dL. However, the job of the mother, exposure to paint or soil, parental education, history of abusing opium, and history of smoking were not significant. The results showed that per one unit of increase in age, the chance of an elevated BLC decreased by 3% (OR (95%CI): 0.97 (0.96–0.99), *p* < 0.01). Further, per one rate of increase in the BMI, the chance of an elevated BLC was higher (OR (95%CI): 1.13 (1.02–1.24), *p* = 0.01). In children aged 12–24 months, the chance of an elevated BLC was 3.87 times that of children older than 72 months (OR (95%CI):3.87 (1.39–10.79), p = 0.01). The risks of an elevated BLC was 61% lower in girls compared to boys (OR (95%CI): 0.39 (0.17–0.92), *p* = 0.03). Finally, in children whose fathers were laborers, the chance of high lead concentrations was 5.56 times higher than those whose fathers were employees (*p* = 0.01) (Table 4).

**Table 4:** logistic regression analysis on the association between blood lead concentrations of children and demographic variables

Variable	OR (95%CI)	*P*-value
Age (month)	0.97 (0.96–0.99)	0.01
Age		
≥ 72 month	Reference	
12–24 month	3.87 (1.39–10.79)	0.01
24–48 month	1.67 (0.56–4.59)	0.35
48–72 month	0.62 (0.16–2.47)	0.50
BMI (kg/m^2^)	1.13 (1.02–1.24)	0.01
Gender		
Boy	Reference	
Girl	0.39 (0.17–0.92)	0.03
Exposure to paint		
No	Reference	
Yes	1.71 (0.69–4.23)	0.25
Exposure to soil		
No	Reference	
Yes	0.53 (0.12–2.33)	0.40
Education levels of parents		
Primary school	Reference	
Middle School	0.57 (0.17–1.92)	0.37
Diploma	0.49 (0.18–1.33)	0.16
Bachelor	0.29 (0.08–1.03)	0.05
Occupation of father		
Employment	Reference	
Martial	4.64 (0.87–24.51)	0.07
Laborer	5.56 (1.44–21.40)	0.01
Repairman	0.03 (0.001–2.65)	0.97
Driver	0.22 (0.08–4.68)	0.80
Self-employment	3.26 (0.89–11.88)	0.07
Occupation of mother		
Self-employment	Reference	
House wife	0.53 (0.11–2.47)	0.42
Employment	0.78 (0.13–4.73)	0.78
Opium use in parents		
No	Reference	
Yes	1.36 (0.17–11.14)	0.77
Cigarette smoking in parents		
No	Reference	
Yes	1.37 (0.30–6.28)	0.68

**Fig. 4:**
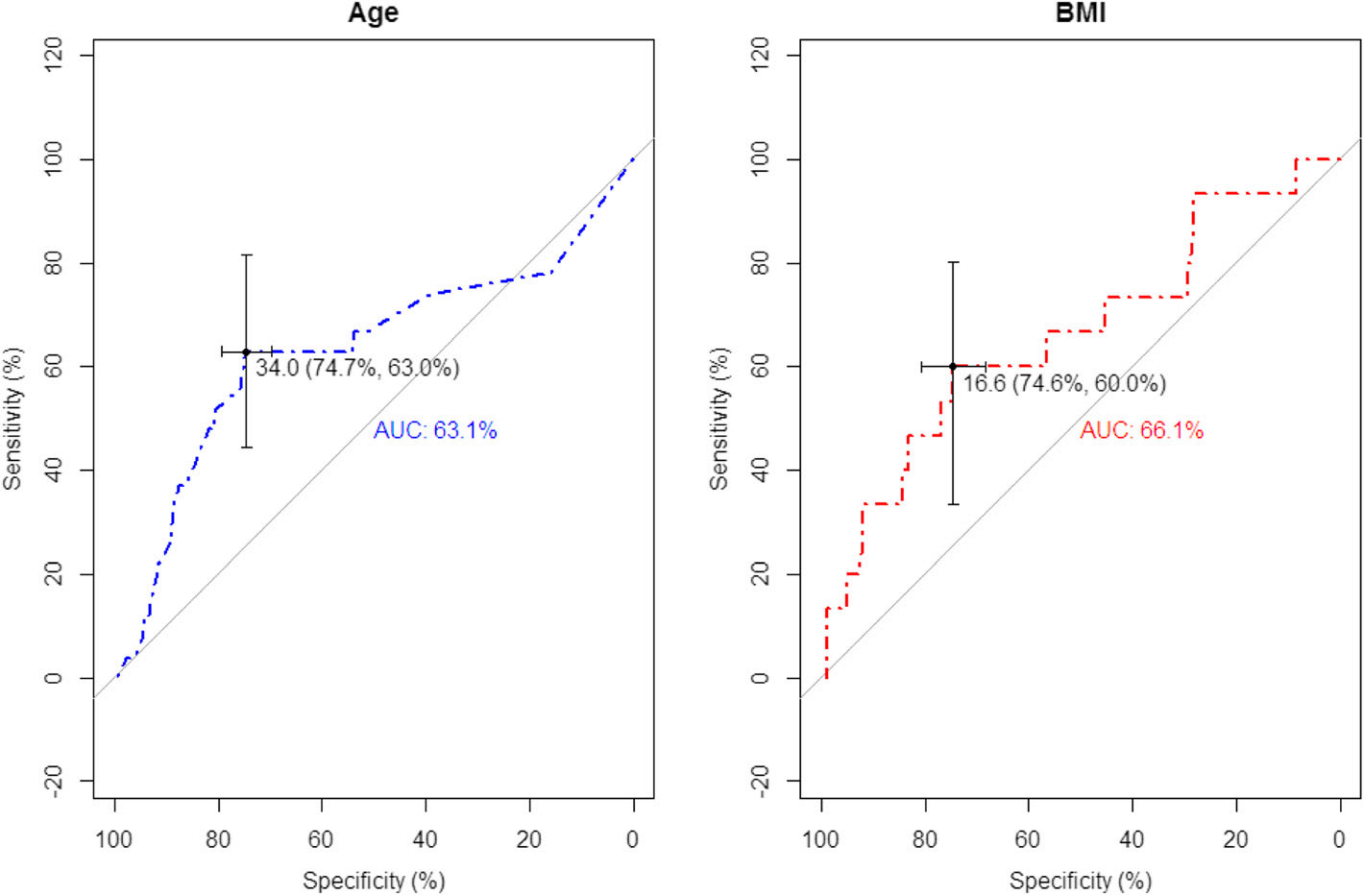
ROC curve for age and BMI in predicting elevated BLC

Based on the ROC curve, the age greater 34.0 months, BMI greater 16.6 kg/m^2^ can predict high blood lead concentrations (≥5 μg/dL) (Fig. 4).

## Discussion

Based on the results of this study, young age significantly increased the chance of high lead concentrations in children. In this regard, some other studies have reported similar results [15–18]. Previous studies also reported that the maximum blood lead concentration rise at the age of around 2 years [15]. These observations are not unexpected, because, at young ages, the child is in the stage of discovering the surroundings by putting their hands or other objects into the mouth (pica). This method of children’s exposure has been well documented in other studies [19, 20]. It has been found that children consume around 50 mg of soil per day on average; if this exceeds 5 g/day, it is known as pica [15]. It has been reported that some children consume 25–60 g/day [21].

The effects of age on elevated BLC need to be more address in future studies.

In children whose fathers were laborers, the chance of elevated BLC was 5.56 times greater than that of employee fathers. Exposure to toxic materials in the workplace is a significant concern for the families of laborers employed in polluting industries [22, 23]. These individuals can carry lead debris and dust from the workplace to the house, thereby exposing the family members to lead [24]. The results of a meta-analysis indicated that around half of the children of workers exposed to lead might have BLC ≥10 μg/dL, and the children of these individuals should undergo blood lead screening [22]. In the study by Chan and Morton, the BLC of children of workers contaminated with lead was higher than that of the control group. In this research, a direct relationship was found between not following professional health instructions and blood lead content [24, 25]. Non-adherence to sanitary and health-related principles by some workers laborers increases the risk of lead exposure. These workers usually neglect precautionary measures such as the use of masks, gloves, and specialized aprons; many of them do not wash their hands before meals and eat food in their workplace [26]. In many developing countries, these stores are in proximity to residential buildings, which increases the risk of exposure of others to lead. Therefore, preventive measures should be taken not only in workers of these occupations but also in the family members and children of these people.

In this study, there was an inverse and significant relationship between blood lead and iron content. Ahamed (2007) tested children with anemia exposed to lead and reported a significant negative correlation between lead and iron concentration [27]. The relationship between high BLC and iron deficiency has also been shown by Muwakkit (2008) and Hegazy (2010) [28, 29]. In these studies, the effect of lead on iron absorption from the digestive system was reported as the reason for iron deficiency. Other investigations, especially in children, have indicated that iron deficiency leads to increased lead absorption from the intestine [30, 31]. Based on the results, boys did not show an iron/lead correlation, while girls with a lower prevalence of elevated blood lead concentrations showed an inverse and significant relationship between lead and iron concentrations. This point may reflect the more susceptibility of girls to lead injuries. Some studies reported that Lead-induced effects on the heme synthesis occur at lower blood lead concentrations in females than in males [32, 33] . Females may also be more prone to neurological and reproductive injuries caused by lead [34].

In this study, the mean BLC of the children was 2.49 μg/dL, well below the CDC reference concentration. However, 8% of studied children had an elevated BLC above 5 μg/dL. Since this age group is most vulnerable to the adverse effects of lead on the nervous system and IQ, preventive interventions should be taken. The reason for the selection of 5 μg/dl as the amount of lead was that the CDC offered the reference BLC as 5 μg/dL or less for children [8]. Studies in other provinces and geographical regions of Tehran reported different results. A study by Daroogar et al. in 2007–2009 showed that the mean BLC of children younger than 10 years old in Tehran was 7.18 μg/dL [35]. Faranoush et al. (2001) performed a study in children 6–11 years old living in Semnan city. In that study, 78.8% of children had BLC > 10 μg/dL and 5% had BLC > 20 μg/dL) [36]. In these studies, the lead concentrations and the prevalence of lead poisoning were much higher than in the children described in our study. It shows these studies were performed in more contaminated locations since Tehran and Semnan are industrial cities with higher air pollution. Besides, In Iran, the leaded gasoline was extensively used until January 2002 [37] and accounted for higher BLC in those studies. Different periods separated by nine and 15 years from the current study, the different age range of studies, and greater exposure to some sources of lead can explain the differences observed in these results. There are some changes in the use of house painter, adding lead components to gasoline, etc. elevated BLC in some participants of our study may be attributed to contamination of soil, grounded water, and some food in Birjand with different types of metals, including lead [10–12]. In this regard, previous studies have shown that the mean concentrations of lead (59.46 mg/kg), copper, zinc, and cadmium in the soils of the Birjand and its suburb area were higher than their concentrations in the crust of the land, indicating the presence of heavy metals in human soils. Also, another study showed that all metals concentrations in groundwater were in compliance with national and international standards and guidelines except lead (0.023 mg / l), which was higher than standard guidelines. Besides, another study evaluated heavy metal concentrations and offal samples of cows and sheep in the Birjand area, and they found that all samples were contaminated with lead, cadmium, chromium, copper, and nickel. Extensive studies worldwide have dealt with examining the BLC in children. Similar to this study, Kennedy (2016) reported that between 2013 and 2016 in Flint, Michigan, 3% of children had BLC > 5 μg/dL [38]. Moreover, a national U.S. study (2016) covering the period of 2009–2015 showed that 3% of children younger than 6 years had BLC > 5 μg/dL [17]. In contrast, the BLC of children in Latin America and Caribbean countries between 2000 and 2014 ranged from 25 to 43.2 μg/dL [18], which was much higher than our study. Another study evaluated 28,427 refugee children in the U.S. between 2010 and 2014 (The top 5 overseas examination countries by arrivals were Thailand, Nepal, Malaysia, Iraq, Kenya) and reported an elevated BLC (> 5 μg/dL) in 19.3% of cases, which was higher than our study [16].

The difference observed between different regions, and countries can be attributed to the different food, environmental conditions, and differences in the use of traditional drugs as well as cosmetics (such as Kohl), smoking, and drug abuse. All of these studies highlight the need to constantly investigating the BLC in this vulnerable population.

## Limitations

In this study, we tried our best to investigate the associated factors of elevated BLCs in young children living in Birjand, Iran. But some other potential sources of lead may be involved that were not assessed in this study. This warranted further studies. Since our study investigated the risk factors and consequences concurrently because of its cross-sectional design, this prevented precise investigation of the precedence between them. Prospective studies with a larger sample size are suggested to discover potential factors of elevated blood lead concentrations among children and guarantee the generalizability of the findings to an unselected sample. Also, we did not assess the birth history of included children. To improve the accuracy of results, more extensive data sets, and enhanced data collection methods are suggested in the future. A comparative study for evaluating BLCs in different groups of children aged 1–7 years, infants and older children with considering clinical features and treatment is needed in the future. A follow-up study can be useful to evaluate the trend of lead concentration in the whole blood of children.

## Conclusion

The mean BLC of 1–7-year-old children in Birjand city was 2.49 μg/dL; around 8% of studied children had elevated BLC. Young age, male gender, and low BMI significantly increased the chance of an elevated BLC. These results can provide information for parents with high-risk children to have more attention and doing countermeasures. In children whose fathers were laborers, the risk of high BLC was more significant compared to those children of employee fathers, suggesting ‘take-home’ lead sources at work. Exposure to toxic materials in the workplace is a substantial concern for the families of laborers employed in polluting industries, needing educating workers, and conducting preventive measures. Knowing the BLC in children offers necessary information to health policymakers for public health measures, preventive interventions in the community, and health promotion of children. It also provides baseline data for future researches.

## Supplementary information

**Additional file 1.**

## Data Availability

The datasets are available from the corresponding author on a formal and logical request.

## Abbreviations

BLC: Blood lead concentrations
AAS: Atomic absorption spectrometry
OR: Odds ratio
IQ: Intelligence quotient
CDC: Centers for Disease Control
CBC: Complete blood count
HCT: Hematocrit
HGB: Hemoglobin
MCV: Mean corpuscular volume
