# Supplementary material for: Blood lead concentration and its associated factors in preschool children in eastern Iran: a cross-sectional study
Source: BMC Pediatr. 2020 Sep 16;20:435. doi: 10.1186/s12887-020-02302-7 (PMC7493850; doi:10.1186/s12887-020-02302-7)
Supplement: Supplementary file 1 — Additional file 1. [file 12887_2020_2302_MOESM1_ESM.docx]

Code…….. Date……..

The following questionnaire intends to investigate risk factors of heavy lead exposure in your child. The information will be kept confidential, and there is no need to mention the first or surname. All tests will be done freely.

Please read the questions carefully and mark the correct response.

Phone No.:………….

Gender: Female Male

order of the child in the family:

Age: Weight: Height: BMI:

Birth weight: birth height: head circumference:

Father’s education: mother’s education: number of family members:

Father’s occupation: mother’s education:

Place of residence:

1. Does your child have any known diseases? Yes No

Does your child have any history of allergy? Yes No

The type of known allergy (allergic rhinitis as well as frequent nasal congestion – eczema and itching – asthma and dyspnea – seasonal allergy)?

If the kid is using a drug for allergy treatment, how the kid use the drug? Oral topical

Has your child had any history of thyroid disease? Yes No

Has your child had any history of diabetes? Yes No

Has your child had any history of convulsions? Yes No

2. Has the child begun talking at his average age? Yes No

3. Any history of specific maternal disease? Yes No

Has the mother had a history of gestational diabetes during pregnancy?

Has the mother had a history of gestational hypertension during pregnancy?

Has the mother or father had any history of thyroid disease?

4. History of genetic disease of the father and mother: Yes No

5. Is there any factory or mine close to your place of residence?

Yes Name it……… No

6. Is any of your family members dealing with the following occupations?

Lead processing working in battery-making factory building painting

Welding working in plastic production factory working in a paint factory

Working in mine History of working with vehicles (straightening, exhaust, etc.)

Contact with fuels (gasoline, oil, etc.) Contact with polluted air (police, parking guards, working in terminals – construction activities, and others who work in an open air for a long time…)

7. Do you use glazed earthenware at home? Yes no

8. Are you used to eating soil or turbah? Yes no only during childhood

9. Are you used to taking herbal drugs daily or weekly? Yes no

10. Have you recently painted the walls for a house renovation? Yes (I was present in the house in the meantime I was not present in the house in the meantime) No

11. Do you live in an old house? Yes (How old is the house?...........) No

12. Has any member of the family had a high level of lead? Yes No

13. Is your child exposed to cigarette or hookah smoke at the house? Yes No

14. Do you (father/mother) take drugs of abuse? Yes No I quit; since when?

In the case of “yes” or “I quit”: Opium refined opium Industrial (heroin, crack cocaine, Crystal, etc.)

Route of administration: oral inhalation

Daily consumption level: how long have you taken?

15. History of illicit drug consumption by the child? Yes No

Route of administration: oral inhalation

Daily consumption level:

16. Place of residence: City Village

17. Do you use sunscreen cream? Yes SPF: No

18. How many times a week your child consumes red meat? 1-2 times 3-4 times every day

19. How many times a week do you eat red meat? 1-2 times 3-4 times every day

20. Does your child consume vitamin D supplements? Yes (Dose: ) No

21. Does the child’s mother use a vitamin D supplement? Yes (Dose: ) No

22. Does the child consume milk and dairies regularly?

Thank you, and good luck.
